# Zika virus serologic responses during pregnancy in a dengue-endemic setting: A prospective cohort study in Thailand

**DOI:** 10.1371/journal.pntd.0014805

**Published:** 2026-10-15

**Authors:** Salin Sirinam, Dennopporn Sudjai, Supawat Chatchen, Watcharee Arunsodsai, Weerawan Hattasingh, Laura Pezzi, Thomas Jaenisch, May Chu, Thitiya Yakasaem, Kriengsak Limkittikul

**Affiliations:** 1 Department of Tropical Pediatrics, Faculty of Tropical Medicine, Mahidol University, Bangkok, Thailand; 2 Department of Obstetrics and Gynecology, Rajavithi Hospital, Ministry of Public Health, Bangkok, Thailand; 3 National Reference Center for Arboviruses, Inserm-IRBA, Marseille, France; 4 Unité des Virus Émergents, UVE, Aix-Marseille University, Università di Corsica, IRD 190, Inserm 1207, IRBA, Marseille, France; 5 Center for Global Health, Colorado School of Public Health, University of Colorado, Aurora, Colorado, United States of America; International Centre for Genetic Engineering and Biotechnology, INDIA

## Abstract

**Background:**

Zika virus (ZIKV) infection during pregnancy has been associated with adverse birth outcomes, including severe neurological abnormalities. Confirmation of infection remains challenging due to the disease’s predominantly asymptomatic or mild nature. While real-time reverse transcription polymerase chain reaction (RT-qPCR) is the gold standard, its use in clinical settings and surveillance is limited. Serological point-of-care tests offer alternatives but pose interpretation challenges in Flavivirus-endemic regions.

**Methods and Findings:**

We conducted a prospective cohort of 199 pregnant women attending the antenatal clinic at Rajavithi Hospital, Bangkok, Thailand, in 2021–2022 to describe the incidence of ZIKV serologic responses detected by a rapid test and associated pregnancy outcomes. Participants were monitored using the DPP ZCD IgM/IgG (Zika/Chikungunya/Dengue IgM/IgG) System rapid immunochromatography test (Chembio), with RT-qPCR and plaque reduction neutralization testing performed for further assessment. A ZIKV serologic response was defined as a reactive ZIKV immunoglobulin M (IgM) result at enrollment or seroconversion from non-reactive to reactive ZIKV IgM or IgG between study visits. The DPP ZCD rapid test revealed incident ZIKV-reactive serologic responses at 15.4 per 100 person-years, with all affected participants being asymptomatic. All RT-qPCR results were negative, and participants with ZIKV IgG seroconversion had neutralizing antibodies against ZIKV and all 4 Dengue virus (DENV) serotypes. A prior history of DENV infection was associated with increased odds of ZIKV-reactive serologic response (OR 3.53, *P* value 0.046).

**Conclusion:**

This prospective study identified serologic responses detected by a ZIKV-reactive rapid test among pregnant women in Thailand. In a highly dengue-immune setting, these responses could reflect true ZIKV exposure, DENV reinfection, flavivirus immune boosting, or serologic cross-reactivity, and therefore cannot be attributed specifically to ZIKV. Rapid serologic testing may support screening and surveillance but cannot establish ZIKV infection or ZIKV-specific exposure without careful interpretation and additional testing.

## Author summary

Zika virus infection during pregnancy is a major public health concern because it can cause severe brain defects in fetuses and fetal loss. Detecting this virus is difficult because most infected mothers do not show specific symptoms. Furthermore, standard blood tests often miss the short window when the virus is active in the blood, or takes too long to establish in clinical settings. In this study, we monitored pregnant women in Bangkok, Thailand, using a simple rapid blood test that detects antibodies reactive with Zika virus. We found new serologic responses during pregnancy, but all participants were asymptomatic and RT-qPCR results were negative. In addition, participants with Zika-reactive antibody responses also showed broad antibody responses to Dengue virus. Therefore, the observed responses could represent Zika virus exposure, Dengue reinfection, immune boosting, or cross-reactivity between related flaviviruses. These findings show that rapid serologic tests may be helpful for screening, but results must be interpreted cautiously in regions where Zika and Dengue viruses co-circulate.

## Introduction

Zika virus (ZIKV), a mosquito-borne Flavivirus, was first identified in Uganda in 1947 [1]. After decades of sporadic cases in Africa and Asia, the first significant outbreak officially reported occurred on the Yap Islands (Micronesia) in 2007, followed by emergence in French Polynesia in 2013–2014 [2,3]. In 2015, ZIKV spread to Brazil, marking the start of a major epidemic in the Americas [4]. Shortly thereafter, a sharp increase in microcephaly cases among newborns was observed, leading to the discovery of the link between maternal ZIKV infection and congenital malformations, mainly neurological abnormalities [5–7]. Given its rapid dissemination on a large-scale and severe outcomes, the World Health Organization (WHO) declared ZIKV infection a Public Health Emergency of International Concern in early 2016 [8].

ZIKV infection is usually asymptomatic or mild. Symptomatic cases may present with fever, conjunctivitis, rash, and body aches. Rare but severe neurological complications may occur, notably Guillain-Barré syndrome [9]. Of particular concern are infections occurring during pregnancy, as ZIKV has been associated with congenital zika syndrome (CZS), characterized by microcephaly and other brain abnormalities in the fetus [10]. The risk and severity of fetal abnormalities appear to be correlated with the gestational age: infections occurring during the first and second trimester carry the highest risk, estimated at 6–8% [11]. The infants born to ZIKV-exposed pregnancies may initially remain asymptomatic and present normal head circumference, but later exhibit hidden brain anomalies or neurological impairment detected through follow-up imaging and clinical examinations/assessment [12,13].

Therefore, accurate and timely diagnosis, especially for pregnant women is essential to enable early detection and appropriate management; however, diagnosis of ZIKV infections remains challenging. The clinical presentation is often non-specific, and many infections are asymptomatic. Moreover, laboratory confirmation relies on molecular and serological assays, both limited by several factors. Real-time reverse transcription polymerase chain reaction (RT-qPCR) is considered the gold standard for confirming acute ZIKV infection, typically performed on blood or urine samples. However, ZIKV viremia is usually low and short-lived, often limited to the first week after symptom onset, resulting in possible false-negative results [14]. Viral RNA tends to persist longer in urine than in blood, slightly extending the diagnostic window. Serological testing, including the detection of ZIKV IgM (immunoglobulin M) or evidence of ZIKV IgG (immunoglobulin G) seroconversion, is used to identify recent or past infections [15,16]. These assays have different formats and include rapid diagnostic tests; they all face significant limitations due to cross-reactivity with other Flaviviruses, particularly dengue virus (DENV), in regions where both viruses co-circulate [17,18]. As a result, the diagnosis of ZIKV infections required a combination of tests, yet remaining difficult to implement routinely in clinical settings.

In Thailand, sporadic ZIKV cases have been reported for decades, typically presenting as mild illness, and there is no universal ZIKV screening program for pregnant people in the country. Between October 2020 and September 2022, the cumulative incidence of confirmed ZIKV infection ranged from 0.08 to 0.26 cases per 100,000 population [19]. Following the relaxation of COVID-19 preventive measures, which had previously limited social interactions, temporarily reduced outdoor mosquito exposure, and restricted access to healthcare [20], the incidence of confirmed ZIKV infections increased during 2023–2024 to 0.88 cases per 100,000 population, with Bangkok reporting the highest number of cases [19]. About 5% of confirmed infections were found in pregnant women, with roughly one-third being asymptomatic. Although most pregnancies with fetal abnormalities were not fully investigated to confirm maternal ZIKV exposure as the underlying cause, some cases of CZS have nonetheless been reported, especially in Bangkok since 2020, including among asymptomatic maternal infections [19]. The surveillance case definition in the country includes patients presenting with maculopapular rash along with fever, joint pain or conjunctival injection, as well as neonates with microcephaly, and patients with symmetrical acute flaccid paralysis [21]. With the primary disease burden concerning mainly fetal outcomes in ZIKV-exposed pregnancies, early detection during gestation is critical to identify potential abnormalities as soon as possible.

Herein, we conducted a prospective cohort study to (i) describe the incidence of ZIKV serologic responses detected by a rapid test among pregnant women attending a tertiary medical center in Bangkok, Thailand, between 2021 and 2022; and (ii) assess the interpretation of these responses in relation to RT-qPCR, neutralizing antibody findings, and DENV serologic responses during pregnancy. The Chembio’s Dual Path Platform (DPP) ZCD IgM/IgG (Zika/Chikungunya/Dengue IgM/IgG) rapid immunochromatographic test [22] was employed in this study.

## Materials and methods

### Ethics

The study protocol was approved by both the Ethics Committee of the Faculty of Tropical Medicine, Mahidol University (protocol TMEC 20–062) and the Human Research Ethics Committee of Rajavithi Hospital (protocol 64007). Written informed consent was obtained from all participants prior to enrollment.

### Study site and setting

This prospective cohort study was conducted between November 2021 and November 2022 in the Department of Obstetrics and Gynecology at Rajavithi Hospital, a major tertiary referral center under Thailand’s Ministry of Public Health. Located in the center of Bangkok, the hospital provides comprehensive medical services across more than 1,200 beds.

### Study population

Eligible participants were healthy pregnant women aged 18 years or older with a gestational age (GA) of less than 24 weeks at the time of enrollment. Exclusion criteria were a previous history of ZIKV infection, immunological or autoimmune diseases, use of immunomodulatory or immunosuppressive therapy, receipt of blood products within the previous three months, known fetal abnormalities or high-risk pregnancy, and any condition preventing compliance with the study procedures and follow-up (Fig 1).

**Fig 1:**
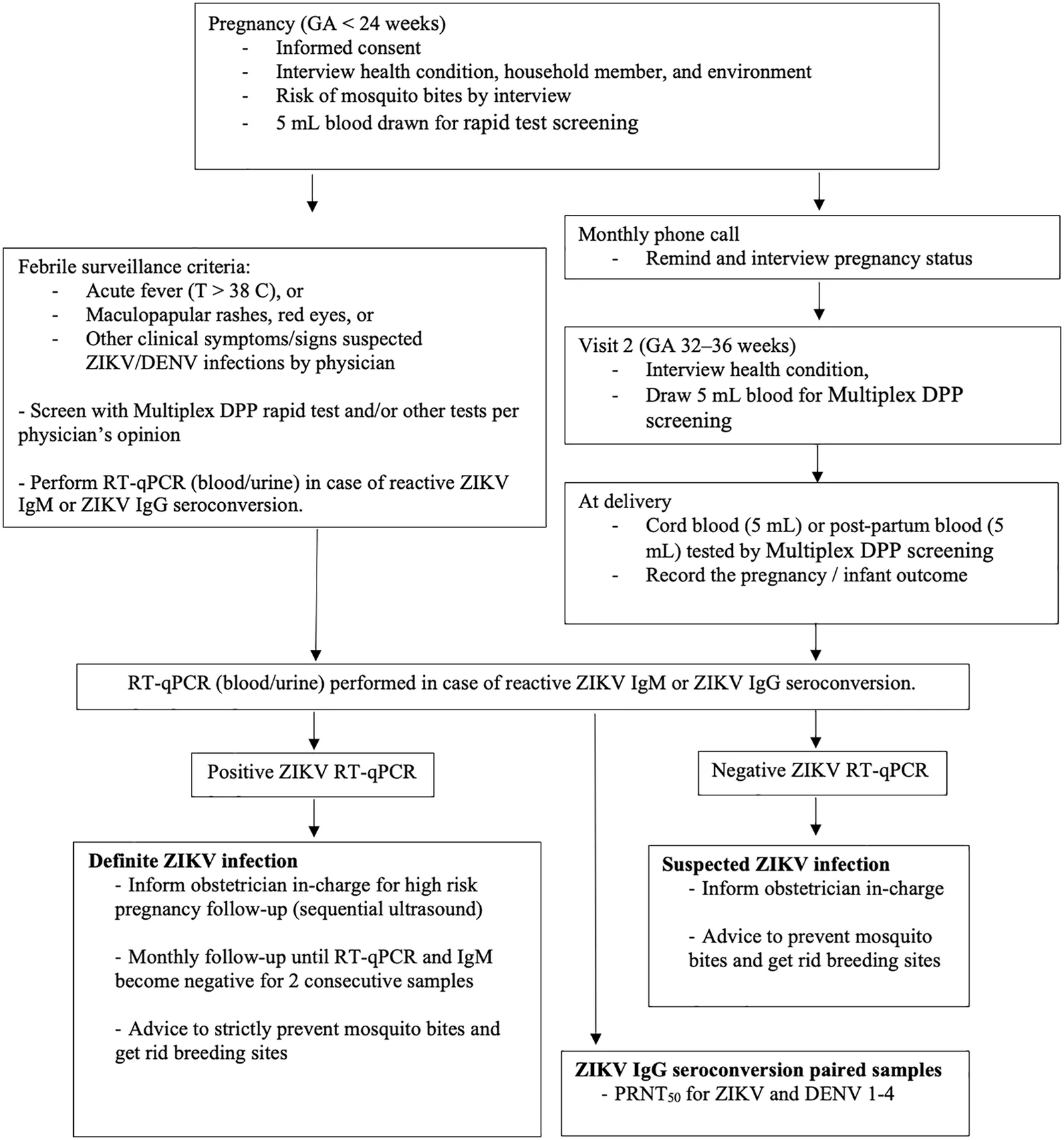
Study design, laboratory testing and ZIKV diagnosis classification.

### Data and samples collection and follow-up

At enrollment, participants completed a standardized questionnaire covering history of febrile illness (specifically ZIKV, DENV, Japanese encephalitis virus, or yellow fever virus) during the preceding six months, as well as any prior flavivirus vaccination. The risk of mosquito exposure within the previous three months was assessed through questions on the average number of daily mosquito bites, household mosquito prevention measures, and awareness of mosquito breeding sites around their residences.

Blood samples were collected at three time points: at enrollment (baseline), at 32–36 weeks of gestation, and at delivery (cord blood) to test with DPP ZCD IgM/IgG System rapid diagnostic test (Fig 1). Pregnancy and neonatal outcomes were recorded for all participants. Surveillance of any febrile presentation was performed until delivery combining passive self-reporting and active follow-up by telephone. Participants who experienced an acute febrile illness with temperature >38 °C, maculopapular rash, or conjunctivitis were asked to provide blood and urine samples for testing with DPP ZCD IgM/IgG System and/or ZIKV RT-qPCR. All samples were analyzed and stored at -80° C at the Department of Tropical Pediatrics, Faculty of Tropical Medicine, Mahidol University.

### Rapid diagnostic tests

The Chembio Dual Path Platform (DPP) ZCD IgM/IgG System (catalog number 65-9565-0) is a single-use rapid immunochromatographic test designed for the qualitative detection of IgM and IgG antibodies against ZIKV, DENV and chikungunya virus (CHIKV) in fingerstick whole blood, EDTA venous whole blood, serum, and EDTA plasma samples. It is intended for point-of-care (POC) use in clinical settings to aid in diagnosis of mosquito-borne viral infections among patients with compatible symptoms or epidemiological exposure.

The device contains upper and lower readout windows with three test lines labeled 1, 2, and 3, corresponding respectively to IgM or IgG antibodies against DENV, ZIKV and CHIKV, along with a control line (C). Testing was performed according to the manufacturer’s instructions. Briefly, results were read 10–15 minutes after buffer addition using the DPP Micro Reader and cassette adapter (catalog number 70-1058-0). Interpretation of IgM and IgG results was performed using the Chembio-developed Radio Frequency Identifier Driven (RFID) DPP ZCD Micro Reader, which verifies the presence of the control line and quantifies the color intensity of each test line, classifying them as reactive (“R”) or non-reactive (“NR”), the latter defined as sera with a numerical intensity value below 22. The absence of a control line indicates that the specimen did not migrate properly along the nitrocellulose strip; the test is therefore considered Indeterminate (“IND”) or Invalid (“INV”) [22].

### Estimation of ZIKV serologic responses

To estimate the incidence of ZIKV serologic responses during pregnancy, both asymptomatic and symptomatic participants were included. A ZIKV serologic response was defined as either a reactive ZIKV IgM result at enrollment (Criterion 1), or seroconversion from non-reactive to reactive ZIKV IgM or IgG results between two visits (Criterion 2 for IgM and Criterion 3 for IgG), as measured by the DPP ZCD IgM/IgG assay. For incidence estimation, new seroconversion events identified by Criteria 2 and 3 were included. Participants meeting these criteria underwent RT-qPCR testing of blood and/or urine, and paired serum samples from participants with ZIKV IgG seroconversion were tested by 50% Plaque Reduction Neutralization Test (PRNT_50_), as described below. Since ZIKV and DENV antibody cross-reactivity may occur in flavivirus-endemic settings, these criteria identify ZIKV-reactive serologic responses and do not establish ZIKV infection or ZIKV-specific exposure.

### ZIKV RT-qPCR

Participants with ZIKV-reactive serologic responses or presentation of symptoms meeting the febrile surveillance criteria (Fig 1) underwent RT-qPCR testing targeting a conserved region of the E gene, performed using qScript XLT 1-Step RT-qPCR ToughMix (Quantabio, MA, USA). The protocol was adapted from a previously published article [23]. Briefly, each 20 µL reaction mixture contained 500 nmol of forward primer (5′- AACATGGCGGAGGTAAGAT-3′), 500 nmol of reverse primer (5′- GTGTCTGATTGCTTGTCAAGGTA-3′), 125 nmol of probe (5′-FAM-AGGCATCAATATCRGACATGGCTTC-BHQ1–3′) and 4 µL of RNA template. Amplification was carried out on a CFX96 Touch Real-Time PCR Detection System (Bio-Rad, CA, USA) under the following cycling conditions: 50 °C for 10 minutes, 95 °C for 1 minute, followed by 45 cycles of 95 °C for 5 seconds and 60 °C for 30 seconds. Each run included a positive (ZIKV (SV0127) virus RNA) and a negative control (nuclease-free water).

### Plaque reduction neutralization test

Paired serum samples from participants with ZIKV IgG seroconversion were tested for specific neutralizing antibodies against ZIKV (strain SV0010/15) and DENV 1–4 (DEN-1 strain 16007, DEN-2 strain 16681, DEN-3 strain 16562, DEN-4 strain C0036/06) using a plaque reduction neutralization test (PRNT) on LLC-MK2 cells, following the 50% plaque reduction criterion as described previously [24,25]. Briefly, a monolayer of LLC-MK2 cells was cultivated in 12-well plates (Corning Life Sciences, Corning, NY, USA). Serum samples were inactivated (56 °C for 30 min) and serially diluted to 1:10, 1:40, 1:160, 1:640, and 1:2560. Each virus was then separately added into diluted serum, and serum–virus mixtures were incubated at 35 °C for 60 min. The mixtures were then inoculated onto LLC-MK2 cell monolayers in 12-well plates. After 4 days of incubation, cells were stained with neutral red. Each serum sample was tested in duplicate, and the number of plaque-forming units was recorded as the mean of two cultures. PRNT_50_ titers were calculated using the probit model, SPSS version 18.0 (SPSS, Inc., Chicago, IL, USA) and expressed as the reciprocal of the last serum dilution causing 50% plaque reduction. A cut-off titer of ≥1:20 was defined as reactive, indicating the presence of neutralizing antibodies.

### Statistical analysis

Sample size estimation was based on an expected number of 150 pregnant women with ZIKV serologic responses over the past three years, assuming an incidence of less than 10%, a 5% margin of error, and a 95% confidence level. The minimum required sample size was 138 participants, increased to 200 participants to account for potential losses to follow-up. Data were analysed using SPSS version 18.0 (SPSS, Inc., IL, USA). Both descriptive and inferential statistical analyses were performed, with results expressed as frequencies, percentages, or rates. Comparisons between groups were made using Chi-square test or Fisher’s exact test for categorical variables (e.g., serological results vs. pregnancy outcomes), and the t-test or Mann-Whitney U test for continuous variables (e.g., antibody titers between seronegative and seropositive participants), as appropriate. Odds ratios (ORs) were calculated to assess associations between demographic characteristics or risk factors and ZIKV-reactive serologic responses. A p-value <0.05 was considered statistically significant.

## Results

### Characteristics of the participants

A total of 199 pregnant women attending the antenatal clinic at Rajavithi Hospital were enrolled in the study at visit 1. Follow-up adherence varied, with participants experiencing pregnancy loss or relocating outside the study area before the subsequent appointments. Overall, 124 participants had serum samples tested at visit 2, and 99 at visit 3. We compared baseline demographic characteristics between participants who remained in the study until visit 3 and those who were lost to follow-up or had an earlier pregnancy loss, and found no statistically significant differences in these variables.

The mean age of participants was 29.6 ± 5.9 years, and the mean gestational age at enrollment was 10.3 ± 4.2 weeks. The majority was gravida (G) 2 (36.2%), followed by G1 (34.7%); 78.9% reported no history of abortion. Thirteen subjects (6.5%) reported underlying medical conditions: 5 with anemia/thalassemia, 2 with peptic ulcer disease, 2 with hypertension and dyslipidemia, 1 with hyperthyroidism, 2 with neurological illness, and 1 with allergic rhinitis. None of the participants declared dengue or yellow fever vaccination, and 16.1% reported a prior DENV infection. Three participants (1.5%) reported a febrile episode within the previous six months, all of whom tested negative for ZIKV IgM (Table 1).

**Table 1:** Demographic data of the participants at the enrollment.

Characteristics		Number (%)
Age at enrollment (years)	18–24	41 (20.6%)
	25–34	114 (57.3%)
	35–44	44 (22.1%)
Gestational age at enrollment (weeks)	<8	70 (35.2%)
	8– < 12	70 (35.2%)
	12– < 16	38 (19.1%)
	16–20	11 (5.5%)
	>20	10 (5.0%)
Gravida	1	69 (34.7%)
	2	72 (36.2%)
	3	39 (19.6%)
	4	16 (8.0%)
	5	3 (1.5%)
Parity	0	90 (45.2%)
	1	69 (34.7%)
	2	32 (16.1%)
	3	8 (4.0%)
Abortion (times)	0	157 (78.9%)
	1	34 (17.1%)
	2	6 (3.0%)
	3	2 (1.0%)
Underlying disease	No	186 (93.5%)
	Yes	13 (6.5%)
Previous infection	COVID-19	27 (13.6%)
	DENV	32 (16.1%)
	Recent acute febrile illness (last 6 months)	3 (1.5%)

At the final follow-up (visit 3), 18 of 199 participants had experienced abortion or stillbirth. Seventeen delivered preterm infants or babies with low birth weight or small for their GA (SGA), while 128 participants had term infants appropriate for GA. The remaining 36 subjects were lost to follow-up for pregnancy outcome assessment. No significant association was found between serological results at any visit and adverse pregnancy outcomes (including abortion/stillbirth, preterm birth or low birth weight) (S1 Table).

### ZIKV-reactive serologic responses and associated factors

A total of 14 participants had ZIKV-reactive serologic responses according to the prespecified criteria: 4 met criterion 1 (reactive ZIKV IgM at enrollment), 1 met criterion 2 (ZIKV IgM seroconversion), 7 met criterion 3 (ZIKV IgG seroconversion) and 2 met both criteria 1 and 3 (reactive ZIKV IgM at enrollment, followed by ZIKV IgG seroconversion). Among the 199 participants enrolled at visit 1, six had reactive ZIKV IgM (3.0%) and 24 had reactive ZIKV IgG (12.1%) at enrollment (Table 2). None of these participants reported an acute febrile episode within the previous six months.

**Table 2:** Baseline ZIKV serostatus at enrollment.

Serological Marker	Result	Number (N = 199)	Prevalence (%)
ZIKV IgM	Reactive	6	3.0
	Non-reactive	193	
ZIKV IgG	Reactive	24	12.1
	Non-reactive	175	

To estimate the incidence of ZIKV-reactive serologic responses, rates were calculated using only the population at risk. Participants who tested reactive at any prior visit were excluded from the denominators of subsequent visits. The percentages shown for visit 2 and 3 therefore represent new serologic responses detected during follow-up. Among at-risk participants with a total follow-up time of 65.03 person-years, we identified 1 ZIKV IgM seroconversion and 9 ZIKV IgG seroconversions. The ZIKV IgM seroconversion occurred between visit 1 and visit 2, corresponding to an IgM seroconversion rate of 0.5% (1 out of 193 at-risk participants). The ZIKV IgG seroconversion rate was estimated at 2.3% (4 out of 175 at-risk participants) between visit 1 and 2, 3.7% (4/107) between visit 2 and 3, and 0.6% (1/175) between visit 1 and 3 (Table 3). Therefore, the incidence rate of ZIKV-reactive serologic responses was 15.4 per 100 person-years. None of the participants with seroconversion reported symptoms compatible with ZIKV infection, and all tested negative for ZIKV RNA by RT-qPCR in blood and/or urine samples.

**Table 3:** Seroconversion rate of ZIKV IgM and IgG during each visit.

SerologicalMarker	Visit 1 (GA <24 weeks)(N = 199)		Visit 2 (GA 32–36 weeks)(N = 124)		Visit 3 (At delivery)(N = 99)	
ZIKV IgM	Reactive	6	Reactive	2	Reactive	1
			Non-reactive	2	Non-reactive	2
			Missing	2	Missing	3
	Non-reactive	193	Reactive	1(0.5% of 193)	Reactive	0
					Non-reactive	0
					Missing	1
			Non-reactive	119	Reactive	0(0.0% of 119)
					Non-reactive	87
					Missing	32
			Missing	73	Reactive	0(0.0% of 73)
					Non-reactive	9
					Missing	64
ZIKV IgG	Reactive	24	Reactive	8	Reactive	2
			Non-reactive	5	Non-reactive	8
			Missing	11	Missing	14
	Non-reactive	175	Reactive	4(2.3% of 175)	Reactive	0
					Non-reactive	2
					Missing	2
			Non-reactive	107	Reactive	4(3.7% of 107)
					Non-reactive	75
					Missing	28
			Missing	64	Reactive	1(0.6% of 64)
					Non-reactive	7
					Missing	56

Among the 7 participants with reactive ZIKV IgM (6 at enrollment and 1 who seroconverted during follow-up), two also had ZIKV IgG seroconversion (participant 1 and 143), three had reactive ZIKV IgG at enrollment (participant 47, 100, and 163), and two remained ZIKV IgG-non-reactive at all visits (participant 70 and 78) (Table 4).

**Table 4:** Serologic profiles and pregnancy outcomes among 14 participants with ZIKV-reactive serologic responses.

Participant code	Visit	GA (week)	DPP result value				Pregnancy outcome
			ZIKV IgM	ZIKV IgG	DENV IgM	DENV IgG	
**Criterion 1: ZIKV IgM-reactive at enrollment**							
1*	1	23	29	NR	NR	26	Term AGA
	2	34	NR	24	NR	28	
	3	Lost F/U	N/A	N/A	N/A	N/A	
47	1	9	26	32	NR	NR	Term AGA
	2	Lost F/U	N/A	N/A	N/A	N/A	
	3	Lost F/U	N/A	N/A	N/A	N/A	
70	1	11	68	NR	NR	NR	Term AGA
	2	32	32	NR	NR	NR	
	3	39	31	NR	NR	NR	
100	1	16	22	100	50	55	Term AGA
	2	32	NR	54	23	26	
	3	39	NR	25	NR	NR	
143*	1	7	35	NR	NR	NR	Term AGA
	2	32	26	NR	NR	NR	
	3	39	NR	33	NR	23	
163	1	5	91	26	NR	23	Abortion
	2	Lost F/U	N/A	N/A	N/A	N/A	
**Criterion 2: ZIKV IgM seroconversion**							
78	1	7	NR	NR	NR	NR	Term AGA
	2	35	120	NR	NR	NR	
	3	Lost F/U	N/A	N/A	N/A	N/A	
**Criterion 3: ZIKV IgG seroconversion**							
1*	1	23	29	NR	NR	26	Term AGA
	2	34	NR	24	NR	28	
	3	Lost F/U	N/A	N/A	N/A	N/A	
6	1	8	NR	NR	NR	35	Term AGA
	2	32	NR	24	NR	61	
	3	Lost F/U	N/A	N/A	N/A	N/A	
36	1	17	NR	NR	NR	NR	Term AGA
	2	34	NR	30	NR	63	
	3	39	NR	NR	NR	44	
98	1	9	NR	NR	NR	NR	Term AGA
	2	32	NR	34	NR	39	
	3	38	NR	NR	NR	NR	
103	1	20	NR	NR	NR	NR	Term AGA
	2	Lost F/U	N/A	N/A	N/A	N/A	
	3	38	NR	28	NR	41	
134	1	5	NR	NR	NR	NR	Term AGA
	2	34	NR	NR	NR	NR	
	3	38	NR	24	NR	32	
137	1	22	NR	NR	NR	NR	Term AGA
	2	36	NR	NR	NR	NR	
	3	39	NR	31	NR	28	
143*	1	7	35	NR	NR	NR	Term AGA
	2	32	26	NR	NR	NR	
	3	39	NR	33	NR	23	
182	1	7	NR	NR	NR	NR	Term AGA
	2	34	NR	NR	NR	NR	
	3	37	NR	27	NR	26	

*Participants 1 and 143 had both reactive ZIKV IgM at enrollment and ZIKV IgG seroconversion.

NR, non-reactive (defined as value <22); AGA, appropriate for gestational age; N/A, not applicable; Lost F/U, lost to follow-up visit.

Among the 9 participants with ZIKV IgG seroconversion during pregnancy, two were also ZIKV IgM-reactive (participant 1 and 143), whereas the remaining 7 either had reactive DENV IgG at enrollment (n = 2) or experienced DENV IgG seroconversion without DENV IgM reactivity (n = 5) (Table 4). Paired sera from participants with ZIKV IgG seroconversion were tested by PRNT_50_: all samples were reactive (cut-off ≥1:20) for neutralizing antibodies against ZIKV and all four DENV serotypes (S2 Table). No statistically significant difference in median PRNT_50_ titers was observed between ZIKV IgG reactive and non-reactive samples (median titer: 632 vs. 637, respectively, p value = 0.30, Mann-Whitney U test). Additionally, no CHIKV IgM or IgG seroconversions were detected by the DPP ZCD IgM/IgG System among participants with ZIKV-reactive serologic response.

Comparisons were made between participants with and without ZIKV-reactive serologic responses to identify factors associated with assay reactivity. Demographic characteristics, including maternal age, gestational age at enrollment, history of abnormal pregnancy, educational level, household monthly income, and presence of underlying disease, did not significantly differ between the two groups. When examining potential risk factors for ZIKV reactivity, participants reporting a prior DENV infection had significantly higher odds of ZIKV serologic response (OR = 3.53, 95% CI 1.02–12.18, p = 0.046). Moreover, approximately 70% of participants without ZIKV serologic responses reported being bitten by mosquito no more than once per day, observing no mosquito larvae in their household area, and regularly using mosquito protection measures. However, no significant associations were found between ZIKV serologic responses and mosquito bites frequency, presence of larvae, or use of mosquito protection methods (Table 5).

**Table 5:** Factors associated with ZIKV-reactive serologic responses.

Factors	ZIKV-reactive, no. (%)	ZIKV-non-reactive, no. (%)	Odds ratio (95% CI)	P- value
Age groups				
• 18–24 years • 25–34 years • 35–44 years	2 (14.3%) 10 (71.4%) 2 (14.3%)	39 (21.1%) 104 (56.2%) 42 (22.7%)	1.48 (0.18–12.17) 2.26 (0.45–11.24) N/A	0.714 0.319
Prior adverse pregnancy outcome*				
• Yes • No	5 (35.7%) 9 (64.3%)	41 (22.2%) 144 (77.8%)	2.14 (0.64–7.20) N/A	0.217
Education				
• > 9 years • ≤ 9 years	11 (78.6%) 3 (21.4%)	134 (72.4%) 51 (27.6%)	1.34 (0.32–5.67) N/A	0.692
Household income per month				
• > 1,000 USD • ≤ 1,000 USD	5 (35.7%) 9 (64.3%)	82 (44.3%) 102 (55.1%)	0.58 (0.17–2.00) N/A	0.387
Underlying disease				
• Yes • No	1 (7.1%) 13 (92.9%)	11 (5.9%) 174 (94.1%)	0.97 (0.10–9.79) N/A	0.978
History of prior DENV infection				
• Yes • No	5 (35.7%) 9 (64.3%)	27 (14.6%) 158 (85.4%)	3.53 (1.02–12.18) N/A	0.046
Frequency of mosquito bite per day				
• More than once a day • 0 − 1	4 (28.6%) 10 (71.4%)	56 (30.3%) 129 (69.7%)	0.81 (0.21–3.09) N/A	0.754
Larvae found in household area				
• Yes • No	6 (42.9%) 8 (57.1%)	56 (30.3%) 129 (69.7%)	1.30 (0.37–4.51) N/A	0.683
Use of mosquito protection measures				
• Yes • No	11 (78.6%) 3 (21.4%)	129 (69.7%) 56 (30.3%)	1.60 (0.40–6.40) N/A	0.507

no., number of participants. USD, United States Dollar. N/A, not applicable.*Adverse pregnancy outcome includes preterm birth, low birth weight, stillbirth, and abortion.

### Pregnancy outcomes in participants with ZIKV-reactive serologic responses

Among the 14 pregnancies with ZIKV-reactive serologic responses, 13 resulted in full-term deliveries of AGA infants, while one ZIKV IgM-positive woman experienced an adverse outcome (Table 4). For this participant (code 163), although PCR was negative, she had a reactive ZIKV IgM result at the enrollment: reactive ZIKV IgM (value 91) at the first visit (GA of 5 weeks), and presence of ZIKV IgG (value 26), with no DENV IgM detected and history of prior DENV infection (DENV IgG-positive). The participant denied any febrile illness within the six months preceding enrollment. Ultrasonographic screening at 12 weeks of gestation was normal; however, at 20 weeks, the fetus was diagnosed with anencephaly, leading to therapeutic abortion. Pathology examination of fetal tissues was not conducted. Overall, the frequency of ZIKV-reactive serologic response did not differ significantly between participants with normal versus adverse pregnancy outcomes (S1 Table). ZIKV serologic response was not significantly associated with adverse pregnancy outcomes (OR 0.26, 95% CI 0.03–2.06, *P* = 0.202). However, the limited number of ZIKV serologic responses renders this analysis underpowered to detect the rare adverse outcomes.

## Discussion

The emergence of ZIKV as a global threat in 2016 and its association with severe congenital defects have prompted sustained interest in its epidemiology and impact during pregnancy. Since most ZIKV infections are asymptomatic or mild, determining the true burden of infection (especially among pregnant women) remains challenging. This longitudinal cohort study demonstrated the incident serologic responses detected by a ZIKV-reactive rapid test among pregnant women in Bangkok in 2021–2022. The observed incidence of these responses was 15.4 per 100 person-years. Given the negative RT-qPCR results, broad neutralizing antibody responses to all 4 DENV serotypes, and concurrent DENV serologic responses, these events cannot be attributed specifically to definite ZIKV infection.

In terms of baseline seroprevalence, 12% of participants had reactive ZIKV IgG at enrollment. In this setting, however, the specific infecting flavivirus cannot be determined from this result alone. In addition, several studies across different regions of Thailand have attempted to estimate ZIKV seroprevalence. A study conducted in 2018 in southern Thailand reported that 12.8% of pregnant people had neutralizing antibody titers (PRNT_90_) ≥10 eighteen months after a ZIKV outbreak [26]. Another study employing a commercial anti-ZIKV IgG ELISA found 30% positivity among pregnant women attending an antenatal clinic in Bangkok in 2019, while 39% of participants exhibited neutralizing antibodies titers ≥10 by focal reduction neutralization test (FRNT_50_) against ZIKV; however, no correlation was observed between ZIKV IgG and ZIKV neutralizing antibodies [27]. The variable levels of ZIKV serologic responses observed through all these studies likely depend on setting, assay performances and study period. In addition, one study in northeastern Thailand found that only half of 0.36% acute ZIKV infections detected by RT-PCR were symptomatic [28]. Together, these studies confirm a ZIKV circulation in Thailand at low but detectable levels and emphasize the predominantly subclinical nature of ZIKV infection or serologic response during pregnancy.

Participants reporting prior DENV infection had 3.5 times higher odds of subsequent ZIKV serologic responses. However, this association should not be interpreted as increased susceptibility to ZIKV infection. Instead, it may reflect overlapping vector exposure in the area, and be influenced by diagnostic cross-reactivity and immune boosting rather than a true increase in susceptibility to acquiring ZIKV. Cross-reactivity among flaviviruses (DENV and ZIKV share extensive antigenic similarity) significantly complicates the interpretation of serological results; in areas where DENV is hyper-endemic (like western Thailand, where more than 98% of adults over 25 years have been reported to be DENV-seropositive [29]), the likelihood of such interference is high. In our study, all sera from participants with ZIKV IgG seroconversion also exhibited antibodies against all four DENV serotypes by PRNT_50_, and seven of nine demonstrated concomitant DENV IgG seroconversion, complicating the determination of the etiological agent. Accordingly, the estimate of 15.4 per 100 person-years should be interpreted as the incidence of ZIKV-reactive serologic responses, not as a ZIKV-specific infection incidence estimate.

Within this complex epidemiological and immunological landscape, ZIKV IgM results on a single sample should be interpreted cautiously. On one side, in case of a ZIKV IgM-positive result, follow-up IgG testing is advised when possible. On the other side, an IgM-negative result does not necessarily rule out ZIKV infection: in fact, ZIKV infections can act as secondary flavivirus infections, resulting in lower IgM titers and a delayed IgM response compared to primary flavivirus infections [30]. A study in the Mexican population, where DENV seroprevalence typically ranges from 70% to 85%, found that IgM positivity in DENV-naive patients reached 86% by the second week of illness, compared to only 33% IgM positivity in DENV-exposed patients [31]. Rapid diagnostic tests such as the DPP ZCD System may be useful for initial screening or surveillance. However, in DENV-endemic settings, a ZIKV-reactive result cannot establish ZIKV infection. Results require cautious interpretation alongside clinical timing and confirmatory assays such as neutralization tests (serology gold standard) and RT-qPCR, to improve diagnostic interpretation.

This study has several limitations. First, although the longitudinal design allowed the detection of incident ZIKV serologic responses, the sample size was relatively small and limited to a single tertiary center in Bangkok, which may not fully represent the broader Thai population or regional variations in ZIKV circulation. Moreover, the sample size is likely underpowered to detect rare outcomes, and the ZIKV IgM-positive pregnancy resulting in fetal loss was not virologically confirmed. Consequently, this adverse outcome is presented descriptively and its interpretation precludes causal inference. Therefore, the association between ZIKV-reactive serologic responses and adverse pregnancy outcome requires further assessment. However, while most newborns in this study appeared healthy at birth, we did not perform long-term neurodevelopmental follow-up. The possibility of delayed sequelae or subtle neurological impairments cannot be excluded. Second, while no RT-qPCR ZIKV-confirmed cases were detected through febrile surveillance, molecular confirmation was primarily performed among participants with ZIKV-reactive serological responses, which offers a limited diagnostic window. Given the short duration of ZIKV viremia and the complexity of antibody responses in DENV-endemic areas, it may not elicit a strong IgM response. Some acute flavivirus infections may have been missed, leading to an underestimation of incidence. Third, the DPP ZCD IgM/IgG rapid test may have limited specificity due to cross-reactivity with dengue antibodies. This issue persisted even with confirmatory PRNT_50_ testing, emphasizing that the rapid serological assays could not distinguish ZIKV-specific infection from DENV reinfection, immune boosting, or cross-reactive flavivirus antibody responses. Fourth, environmental exposure variables, including mosquito bites frequency and protective behaviors, as well as recent febrile episodes, were self-reported and may have been subject to recall bias. The null associations should be interpreted with caution.

## Conclusion

This prospective cohort study documented incident serologic responses detected by a ZIKV antibody rapid test among pregnant women in Bangkok in 2021–2022. In this highly DENV-immune population, negative RT-qPCR findings, concurrent DENV serologic responses, and broad neutralizing antibody responses to DENV precluded the diagnosis of ZIKV infection and highlight the challenge of serological diagnosis in DENV-endemic regions. Therefore, rapid serologic assays may support screening and surveillance during pregnancy, but they should not be used alone to establish ZIKV infection in flavivirus-endemic settings. Larger studies integrating molecular and serological methods are needed to better quantify incidence and assess the risk of adverse pregnancy outcomes in pregnant women with ZIKV serologic responses.

## Supporting information

S1 Table Comparison between serological results and pregnancy outcomes. (DOCX)

S2 Table PRNT_50_ titer of ZIKV IgG seroconverted paired sera. (DOCX)
