# Supplementary material for: Zika virus serologic responses during pregnancy in a dengue-endemic setting: A prospective cohort study in Thailand
Source: PLoS Negl Trop Dis. 2026 Oct 15;20(10):e0014805. doi: 10.1371/journal.pntd.0014805 (PMC13712765; doi:10.1371/journal.pntd.0014805)
Supplement: Supplementary file 1 [file pntd.0014805.s001.docx]

**Supplementary Table 1. Comparison between serological results and pregnancy outcomes.**

| **Visit** | **Serology** | **Result** | **Pregnancy outcome (no.)** | | ***P* value**** |
| --- | --- | --- | --- | --- | --- |
|  |  |  | **Adverse*** | **Normal** |  |
| 1 | ZIKV IgM | Reactive | 1/35 (2.9%) | 5/128 (3.9%) | 0.619 |
|  |  | Non-reactive | 34/35 (97.1%) | 123/128 (78.3%) |  |
|  | ZIKV IgG | Reactive | 4/35 (11.4%) | 14/128 (10.9%) | 0.570 |
|  |  | Non-reactive | 31/35 (88.6%) | 114/128 (89.1%) |  |
| 2 | ZIKV IgM | Reactive | 0/14 (0.0%) | 3/99 (3.0%) | 0.670 |
|  |  | Non-reactive | 14/14 (100.0%) | 96/99 (97.0%) |  |
|  | ZIKV IgG | Reactive | 1/14 (7.1%) | 9/99 (9.1%) | 0.641 |
|  |  | Non-reactive | 13/14 (92.9%) | 90/99 (90.9%) |  |
| 3 | ZIKV IgM | Reactive | 0/16 (0.0%) | 1/83 (1.2%) | 0.838 |
|  |  | Non-reactive | 16/16 (100%) | 82/83 (98.8%) |  |
|  | ZIKV IgG | Reactive | 1/16 (6.3%) | 6/83 (7.2%) | 0.685 |
|  |  | Non-reactive | 15/16 (93.8%) | 77/83 (92.8%) |  |
| Total | ZIKV infection | Yes | 1/35 (2.9%) | 13/128 (10.2%) | 0.152 |
|  |  | No | 34/35 (97.1%) | 115/128 (89.8%) |  |

no., number of participants

*Adverse pregnancy outcome includes preterm birth, low birth weight, stillbirth, and abortion.

**Fisher’s Exact Test
