# Supplementary material for: Zika virus serologic responses during pregnancy in a dengue-endemic setting: A prospective cohort study in Thailand
Source: PLoS Negl Trop Dis. 2026 Oct 15;20(10):e0014805. doi: 10.1371/journal.pntd.0014805 (PMC13712765; doi:10.1371/journal.pntd.0014805)
Supplement: Supplementary file 2 [file pntd.0014805.s002.docx]

**Supplementary Table 2. PRNT_50_ titer of ZIKV IgG seroconverted** **paired sera.**

| **Code** | **Visit** | **GA (week)** | **DPP ZCD IgM/IgG System** | | | | | **PRNT_50_ titer** | | | | |
| --- | --- | --- | --- | --- | --- | --- | --- | --- | --- | --- | --- | --- |
|  |  |  | **ZIKV IgM** | **ZIKV IgG** | **DENV IgM** | **DENV IgG** | **ZIKV** | | **DENV-1** | **DENV-2** | **DENV-3** | **DENV-4** |
| 1 | 1 | 23 | 29 | NR | NR | 26 | 637 | | 323 | 885 | 297 | 666 |
|  | 2 | 34 | NR | 24 | NR | 28 | 632 | | 620 | 1133 | 249 | 846 |
|  | 3 | Lost F/U | N/A | N/A | N/A | N/A | N/A | | N/A | N/A | N/A | N/A |
| 6 | 1 | 8 | NR | NR | NR | 35 | 1342 | | >2560 | >2560 | >2560 | 1097 |
|  | 2 | 32 | NR | 24 | NR | 61 | >2560 | | >2560 | >2560 | 2072 | 1291 |
|  | 3 | Lost F/U | N/A | N/A | N/A | N/A | N/A | | N/A | N/A | N/A | N/A |
| 36 | 1 | 17 | NR | NR | NR | NR | 51 | | 577 | 1714 | 552 | 1040 |
|  | 2 | 34 | NR | 30 | NR | 63 | 11 | | 428 | 1058 | 259 | 857 |
|  | 3 | 39 | NR | NR | NR | 44 | N/A | | N/A | N/A | N/A | N/A |
| 98 | 1 | 9 | NR | NR | NR | NR | 51 | | 1181 | 1334 | 492 | 698 |
|  | 2 | 32 | NR | 34 | NR | 39 | 48 | | 1551 | 1321 | 646 | 509 |
|  | 3 | 38 | NR | NR | NR | NR | N/A | | N/A | N/A | N/A | N/A |
| 103 | 1 | 20 | NR | NR | NR | NR | >2560 | | 291 | 1554 | 247 | 251 |
|  | 2 | Lost F/U | N/A | N/A | N/A | N/A | N/A | | N/A | N/A | N/A | N/A |
|  | 3 | 38 | NR | 28 | NR | 41 | >2560 | | 207 | 1720 | 158 | 335 |
| 134 | 1 | 5 | NR | NR | NR | NR | N/A | | N/A | N/A | N/A | N/A |
|  | 2 | 34 | NR | NR | NR | NR | 166 | | 224 | 593 | 758 | 613 |
|  | 3 | 38 | NR | 24 | NR | 32 | 305 | | 338 | 593 | 401 | 658 |
| 137 | 1 | 22 | NR | NR | NR | NR | N/A | | N/A | N/A | N/A | N/A |
|  | 2 | 36 | NR | NR | NR | NR | 1396 | | 1162 | 1373 | 689 | 1151 |
|  | 3 | 39 | NR | 31 | NR | 28 | 809 | | 1302 | 1174 | 728 | 1228 |
| 143 | 1 | 7 | 35 | NR | NR | NR | N/A | | N/A | N/A | N/A | N/A |
|  | 2 | 32 | 26 | NR | NR | NR | 227 | | 1755 | 1380 | 1016 | 1160 |
|  | 3 | 39 | NR | 33 | NR | 23 | 70 | | 1968 | 1464 | 992 | 927 |
| 182 | 1 | 7 | NR | NR | NR | NR | N/A | | N/A | N/A | N/A | N/A |
|  | 2 | 34 | NR | NR | NR | NR | 1634 | | 127 | 901 | 70 | 75 |
|  | 3 | 37 | NR | 27 | NR | 26 | 1115 | | 153 | 879 | 67 | 219 |

NR, non-reactive; AGA, appropriate for gestational age; N/A, not applicable; Lost F/U, lost follow-up visit.
